# Predictors of job satisfaction among teachers in Germany during the SARS-CoV-2 pandemic: cross-sectional results of a nationwide online questionnaire

**DOI:** 10.3389/fpsyg.2023.1168647

**Published:** 2023-05-26

**Authors:** Theresa Dicks, Viktoria Eggert, Clemens Koestner, Carolina Zähme, Till Beutel, Kristin Kalo, Stephan Letzel, Pavel Dietz

**Affiliations:** ^1^Institute of Occupational, Social and Environmental Medicine, University Medical Center of the University of Mainz, Mainz, Germany; ^2^Institute for Teachers’ Health, University Medical Center of the Johannes Gutenberg University of Mainz, Mainz, Germany; ^3^Department of Sports Medicine, Disease Prevention and Rehabilitation, Johannes Gutenberg University Mainz, Mainz, Germany

**Keywords:** SARS-CoV-2, Germany, COVID-19, teachers, job satisfaction, work-related predictors, sociodemographic predictors

## Abstract

**Introduction:**

During the SARS-CoV-2 pandemic teaching was changed several times to distance learning. To consider the associated stresses and challenges for teachers a nationwide cross-sectional study was performed in March 2021 in which *N* = 31,089 teachers from Germany participated.

**Methods:**

A multiple linear regression model with stepwise inclusion of thematically sorted variables (sociodemographic, SARS-CoV-2- and work-related variables) was used to identify relevant predictors of job satisfaction.

**Results:**

The analysis revealed that work-related variables were significant predictors of job satisfaction. In the third regression model, when all variables are included the adjusted *R*^2^ was 0.364. Overall, the results showed that, e.g., work predictability (*b* = 0.097), influence at work (*b* = 0.118), and meaning of work (*b* = 0.212) increased job satisfaction. In contrast, increased emotional exhaustion (*b* = −0.016), feelings of unfair treatment (*b* = −0.048), and work family conflicts (*b* = −0.154) deteriorated job satisfaction.

**Discussion:**

The results indicate that future research should focus especially the work-related topics in more detail and that job satisfaction is a useful concept for analyzing working conditions from a public health point of view.

## 1. Introduction

### 1.1. Job satisfaction – theoretical background

There are many different definitions of job satisfaction in relevant literature, depending on the focus of each analysis. The analyses in the present paper refer to general job satisfaction, which describes how satisfied individuals are with their job in general (Ali et al., 2014).

In addition to specific recommendations for measuring job satisfaction in survey research, some theoretical models describe the predictors of job satisfaction. The classic models of job satisfaction were developed for companies that correspond to a traditional office job (Mache and Harth, 2017). Due to the specific structures underlying the teaching profession, the Hackman and Oldham’s Job Characteristic-Model and the two-factor theory of Herzberg were suitable models for this study, and are therefore used as a theoretical background of this paper.

The Job Characteristics-Model identifies five different work dimensions, which influence individual and work outcomes. The five work dimensions are autonomy, feedback, skill variety, task significance, and task identity, which positively or negatively influence motivation depending on their degree of expression. The three elements of skill variety, task significance, and task identity can be summarized as the meaningfulness of work (Hackman and Oldham, 1974). The score of the three dimensions (autonomy, feedback and meaningfulness of work) results in different personal outcomes (work motivation, job satisfaction, quality of job performance, and possible absenteeism) from which this study refers to the outcome of job satisfaction. The model assumes that autonomy can be measured by experienced responsibilities for work content and feedback in the form of knowledge about current results and work activities. The higher the autonomy and feedback of the employees, the higher the expected personal and professional outcomes (Hackman and Oldham, 1974).

In addition to the Job Characterics-Model, Herzberg’s two-factor theory is frequently used as an explanatory model for job satisfaction (Stello, 2011). The idea is that so-called hygiene factors can prevent dissatisfaction (e.g., salary or job security), but this does not mean that satisfaction is generated. Generating satisfaction requires so-called motivator factors that increase job satisfaction through direct motivation. Such enriching factors include, for example, autonomy, responsibility, or the possibility of further development. Previous research has shown significant relationships between these job characteristics and job satisfaction (Ali et al., 2014). The analysis of Fritz et al. (2011) indicates that several aspects of the theoretical models significantly influence job satisfaction. For example, parts of autonomy, development opportunities, or support from superiors are considered sources of resources. At the same time, on the burden side, role conflicts and emotional stress should be mentioned (Fritz et al., 2011). Following this theory and assuming that stressors and challenges have increased in the context of the SARS-CoV-2 pandemic, in the present paper SARS-CoV-2-specific variables were also considered. These were intended to capture the stressors that arose during the pandemic.

In addition to the classical research models of job satisfaction, role-theoretical aspects will be explained in more detail to improve the understanding of work family conflicts. Role theory offers a starting point for describing the combination of different expectations of action and potential conflicts between other areas of life. The idea of role theory is that situational behavioral expectations are bound to all actors in a society, which can be found in the performance of the role. By taking on several positions in different areas, different role expectations are posed to a person, depending on the context of the respective frame of reference (Greenhaus and Beutell, 1985). In this context, conflicts between different areas of life can arise due to, for example, limited time resources and diverging expectations (Beham et al., 2010). The resulting tensions between the role expectations of the different domains are also referred to as inter-role conflicts. Such inter-role conflicts often occur between work and private life, where limited resources compete. This is often described as a so-called work family conflict (Greenhaus and Beutell, 1985). The problems of reconciling the two areas of life can lead to a deterioration in satisfaction with life in general as well as a decline in job satisfaction. Various studies showed, that work family conflicts worsen job satisfaction and are often related to work demands, time resources, or emotional stress (Cortese et al., 2010; Mache and Harth, 2017).

### 1.2. The importance of job satisfaction and aim of the study

The SARS-CoV-2 pandemic led to changes in the daily organization of work in almost all occupational areas, including schools (Ellis et al., 2020). Such changes in organization-specific characteristics are often related to job satisfaction and supplemented by employee-specific characteristics (Mache and Harth, 2017). During the pandemic, many teachers were forced to work exclusively from home in distance teaching (UNESCO, 2020). Consequently, teachers’ already rarely completed spatial separation of work and private life was further reduced. In line with role theory considerations, this harbors an increased potential for conflicts between work and personal life. Studies have documented a correlation between these two variables – an increased work family conflict often leads to decreased job satisfaction (Erdamar and Demirel, 2016; Mache and Harth, 2017; Göbel et al., 2022). The SARS-CoV-2 pandemic has caused several challenges for the work of teachers. Studies mention methodological or technical problems in planning and delivering lessons. Concerns about children left behind and lack of contacts are also frequently raised (Tadesse and Muluye, 2020; Tarkar, 2020; Winter et al., 2021).

A review of current scientific discussions showed that analyses of job satisfaction were suitable for identifying aspects that affect teachers’ mental health and for deriving concrete recommendations for measures based on them (Nadinloyi et al., 2013; Steinlin et al., 2016). The topic of teachers’ well-being is emphasized in the findings that teachers, in particular, have an increased risk of burnout as well as higher levels of symptoms for depression and generalized anxiety, when compared to the general population and often retire early (Bauer, 2009; Awa et al., 2010; Koestner et al., 2022).

In summary, it appears that job satisfaction is an important measure to capture the well-being of employees. In particular, the theoretical background shows that changes in the organization of work or the private environment can influence this. It can be assumed that numerous structures, that might influence job satisfaction, also changed due to the SARS-CoV-2 pandemic. Few studies address teacher job satisfaction during the SARS-CoV-2 pandemic. Here, particular attention is paid to the fact that the pandemic-related changes in teaching activities also had a negative impact on job satisfaction. The reasons for this are, in particular, the increased workload and the challenges of distant learning (Suganya and Sankareshwari, 2020; Aktan and Toraman, 2022).

Regarding the work environment of teachers in total, there was a knowledge gap about the predictors of job satisfaction especially during the SARS-CoV-2 pandemic. Understanding the conditions and predictors that are associated with job satisfaction during the SARS-CoV-2 pandemic, as well as the conditions and variables that predict job satisfaction were necessary information that contribute to evidence-based planning of strategies to promote job satisfaction. The effectiveness of such strategies is increased when they are adapted to conditions and factors related to job satisfaction. Therefore, these potential predictors of job satisfaction need to be investigated. The present study provides a suitable starting point for assessing teachers’ job satisfaction during the pandemic and identifies possible predictors from various areas. Based on the preliminary consideration, the aim of the present paper was (i) to assess job satisfaction among teachers in Germany during the SARS-CoV-2 pandemic and (ii) to investigate sociodemographic, work-related, and pandemic-specific predictors of teachers’ job satisfaction. In addition to predictors that negatively influence job satisfaction, particular attention will be paid to those that positively influence job satisfaction to derive concrete, practical recommendations, and evidence-based recommendation to improve teachers’ job satisfaction in future.

## 2. Methods

### 2.1. Study design and procedure

A Germany-wide online questionnaire was conducted among teachers between March 1st and March 31st 2021 to assess the burdens, demands and work conditions. In cooperation with the Ministry of Education as well as the Supervisory and Service Directorate (ADD) of the German federal state Rhineland-Palatine, the Education and Science Union (GEW), the German Teachers’ Association, and the Monitor Lehrerbildung project, the online questionnaire was distributed to teachers in Germany and participation was advertised. Participation in the study was voluntary. There was a non-monetary incentive (EUR 2000.00 donation to the German Children’s Fund) to encourage willingness to participate. The study was conducted with the software LimeSurvey, and participants gave informed consent before their participation. Approval to perform the study was given by the ethical committee of the Medical Association of Rhineland-Palatinate (application number: 2020-15,531).

### 2.2. Measures

The online questionnaire covered a wide range of health-, work- and SARS-CoV-2-related topics with approximately 350 items from validated questionnaires and some self-designed or SARS-CoV-2-specific items. The online questionnaire was pretested in several steps by the Institute for Teachers’ Health at the Institute for Occupational, Social and Environmental Medicine of the University Medical Center Mainz, teachers, and our research team.^1^

**Dependent variable**: Haarhaus’s (2016) short questionnaire was used to measure *job satisfaction*. Job satisfaction can be reliably and validly measured by recording it with one item (Haarhaus, 2016). The item used was: “How satisfied are you with your job situation overall?” Participants responded on a five-point scale (1 “Not at all”, 2 “Not very much”, 3 “Quite a bit”, 4 “Very much” 5 “Extremely”).

**Independent variables**: 26 variables were used to analyze their predictive value for job satisfaction after pretesting. They were divided into the following three thematically categorized blocks for the analysis: (1) sociodemographic variables, (2) SARS-CoV-2-specific variables, and (3) work-related variables.

(1) The sociodemographic information was collected from the questionnaire by asking questions about *gender* (“male,” “female,” “diverse”), *age* (recorded in years as a continuous variable), working in *school management* (“yes,” “no”), *working time model* (“full-time,” “part-time”), *school type* (“primary school,” “secondary general school,” “secondary school,” “academic secondary school,” “comprehensive school,” “vocational school,” “special needs school,” “other school types”). All variables at categorical scale level mentioned up to here had mutual exclusive response categories. For the regression the variable *age* was centered at the mean.
(2) The SARS-CoV-2 related variables were collected by different self-constructed items. The *increased private burdens* were measured with the statement “Since the beginning of the SARS-CoV-2 pandemic, private stresses have increased overall in my life.” The respondents could indicate their agreement or disagreement with the statement on a five-point scale: “Strongly disagree” “Disagree” “Partly” “Agree” “Strongly agree”. These response scales were also used for the following other variables: *increased conflicts in household* (“Since the beginning of the SARS-CoV-2 pandemic, there has been increased conflict in my household”), *restrictions in recreational activities* (“Since the beginning of the SARS-CoV-2 pandemic, there have been restrictions in my leisure activities (e.g., sports, club activities, meetings with friends)”), and *economic difficulties* (“The SARS-CoV-2 pandemic is causing myself/my household great economic hardship”). In addition, a variable on Corona-associated fear was also included in Model 2. The variable *SARS-CoV-2-related fears* is a dimension formed by a principal component analysis (PCA), which is composed of four individual question sets about ‘general fear of COVID infection’, ‘fear of severe progression’, or ‘fear of infection of other persons’ or ‘fear of infection of close relatives’. To measure *SARS-CoV-2-related fears*, participants rated the four different items on a scale from 0 (no anxiety) to 100 (high anxiety). Detailed information about the questions of these items can be obtained from the supplementary, the process of the principal component analysis will be described in the next chapter.
(3) Information about the working conditions were collected by using mainly the validated German version of the Copenhagen Psychological Questionnaire (COPSOQ) (Burr et al., 2019; Lincke et al., 2021). The used COPSOQ variables are on the one hand single items: *meaning of work*, *social support colleagues, social support supervisor* and *cannot forget work*. On the other hand, there are scales, which are composed of several questions: *work family conflict*, *predictability*, *influence at work*, *hiding emotions*, *emotional demands*, and *feedback*. To create the scales, the mean value of the individual questions was calculated. Supplementary Table S1 gives an overview of the relevant variables of COPSOQ and their corresponding questions. In additions to the COPSOQ variables a few other variables were included. *Emotional exhaustion* was measured by asking the question “How often do you feel burned out from your work?” (“Never,” “At least a few times a year,” “Min. once a month,” “Some times per month,” “Once a week,” “Multiple times a week,” “Daily”). This is a validated German adaptation of the Maslach Burnout Inventory based on the work of Büssing and Perrar (1992). In addition, a self-created question on *unfair treatment* with a five point response scale was created: “In my everyday working life, I often have the feeling that I am being treated unfairly” (“Strong rejection,” “Rejection,” “Slight disapproval,” “Light agreement,” “Agree,” “Strong agreement”).

### 2.3. Statistical analyses

Descriptive statistics were calculated for the sociodemographic variables to get an overview of the sample. Principal component analysis was performed to reduce similar questions of the SARS-CoV-2 related fears to one common dimension (Kopp and Lois, 2014). The Kaiser-Meyer-Olkin value for the PCA was 0.703 and Bartlett’s tests showed a significance of *p* ≤ 0.001, so the criteria for a PCA were successful (see Supplementary Table S2). The results show that all four variables (“general fear of COVID infection,” “fear of severe disease progression,” or “fear of infecting others” or “fear of infecting close relatives”) can be combined into one component because the loadings of each variable are at least 0.6 (see Supplementary Table S3). The variable formed by the principal component analysis is called *SARS-CoV-2 related fears*. Furthermore, independent variables were selected, using Spearman correlation (see Supplementary Table S4).^2^ Only the variables that showed a significant correlation (*p* ≤ 0.001) with job satisfaction were included in a multivariate linear regression analysis. The selected variables were operationalized in various aspects in preparation for further analysis. In the case of the variable gender, the number of cases in the ‘diverse’ category was very small compared to the other categories (*n* = 139). Therefore, it was excluded from the regression analysis. Regarding the statistical process it is important to notice, that the dependent variable of job satisfaction, as well as a major part of the independent variables were quasi-interval structured. There is controversial discussion in the literature about the use of an ordinal scale as an interval scale. A simulation study, which is referring to this discussion, shows that even ordinal variables with only a few expressions can be considered quasi-interval (Wu and Leung, 2017). In particular, because of the advantages of ordinary least square (OLS) regressions for determining predictors, the variable of job satisfaction is treated as quasi-interval in these analyses. As part of the model specification of the OLS regression and in order to exclude problems caused by only a few categories of the dependent variable, the BLUE assumptions (best linear unbiased estimator) were also observed. The Durbin Watson test was used to assess whether there was a serious scattering inequality among the residuals of the regression analysis. In addition, the variance inflation factor and collinearity matrix were determined to check whether multicollinearity was present or not (Urban and Mayerl, 2018). Across all regression assumptions, no remarkable abnormalities could be detected. After this, the multivariate linear regression analysis with stepwise inclusion of the three variable groups (models) was conducted to identify relevant predictors of job satisfaction. In the context of multivariate data analysis, the independent variables can be added step by step by dividing the data into different models. The stepwise inclusion of variables in a regression is a frequently used procedure, especially for questions about predictors (Johnsson, 1992). The different blocks can be composed by theoretical or, as in this paper, content-based sorting of variables. The procedure helps identify possible mediations between independent variables and provides an accurate estimate for the residuals (Kopp and Lois, 2014). To determine a sufficient sample size, a power analysis (a two-sided alpha-error (*α*) level of 1% and a 95% power) was performed. According to this, a minimum of approximately 9,500 participants was calculated. Statistical analyses were performed using IBM SPSS Version 27.

## 3. Results

### 3.1. Sample characteristics and descriptive measures

A total of 39,359 teachers participated in the online questionnaire. After data cleaning, the sample size was *N* = 31,089.

In the whole sample, 77.5% (*n* = 24,099) of participants reported being female, 22.0% (*n* = 6,851) male, and 0.4% (*n* = 139) diverse. The participants’ age ranged from 18 to 67 years, with an average age of 45.8 years (SD = 10.5). Overall, 60.3% (*n* = 18,662) of the participants were employed full-time, and 39.7% (*n* = 12,297) were employed part-time. The distribution of the individual school types in our online questionnaire can be seen in Table 1. Furthermore, 10.6% (*n* = 3,290) of the participants reported being part of the school management, and 89.4% (*n* = 27,691) did not.

**Table 1:** Sample characteristics.

Variable	Sample characteristics
Gender	31,089 (100%)
Female	24,099 (77.5%)
Male	6,851 (22.0%)
Diverse	139 (0.4%)
Age, mean ± SD (range)	45.8 ± 10.5 (18–67)
School management	30,981 (100%)
Yes	3,290 (10.6%)
No	27,691 (89.4%)
Working time model	30,959 (100%)
Full-time	18,662 (60.3%)
Part-time	12,297 (39.7%)
School type	27,960 (100%)
Primary school	9,030 (32.3%)
Secondary general school	539 (1.9%)
Secondary school	2,162 (7.7%)
Academic secondary school	5,451 (19.5%)
Comprehensive school	4,016 (14.4%)
Special needs school	2,969 (9.6%)
Vocational school	2,699 (9.7%)
Other	1,367 (74.9%)

Regarding the dependent variable of job satisfaction among all participants, around 40% reported being ‘not at all’ or ‘not very much’ satisfied with their job situation. Less than one-fifth of the participants described their job situation as ‘very much’ or ‘extremely’ satisfied.

### 3.2. Predictors of job satisfaction

As the correlation matrix in the Supplementary Table S4 show, a significant relationship was revealed between a large proportion of the independent variables and job satisfaction. The variables ‘secondary general school’, ‘comprehensive school’, ‘other school’, and ‘minor children in household’ were excluded from further analyses as they did not show significant correlation (see Supplementary Table S4).

Since the present multiple linear regression analysis refers to a selection of variables for which there was also the option of answering nothing, the number of cases included into the regression analysis was reduced to *n* = 16,043. This means that only cases were included that did not have a missing on any of the considered variables. Across all variables, variance inflation factors ranged from 1.038 to 1.808. Thus, the assumption of independence of the regressors can be confirmed. The Durbin–Watson value of the full regression model is 1.997, so the assumption that there is no autocorrelation between the residuals can be confirmed.

Table 2 shows the results of the multiple regression analysis after stepwise inclusion of the three models. For better clarity, the independent variables were sorted according to the explained sociodemographic, work-related, and SARS-CoV-2-related predictors, and significant results were marked accordingly.

**Table 2:** Predictors of job satisfaction after stepwise inclusion of the three variable blocks into the multiple linear regression (*N* = 16,043).

	Variable	Model 1	Model 2	Model 3
Sociodemographic variables	Gender (women)	−0.125^*^	−0.076^***^	−0.017
	Age-centered in years	−0.005^***^	−0.007^***^	−0.007^***^
	School management (yes)	0.224^***^	0.173^***^	0.125^***^
	Working time model (full-time)	0.034^*^	0.009^*^	0.040^***^
	Primary school	−0.030	−0.022	−0.070^***^
	Secondary school	−0.090^**^	−0.090^***^	−0.085^***^
	Academic secondary school	0.074^***^	−0.061^**^	0.068^***^
	Vocational school	0.117^***^	−0.113^***^	0.062^**^
	Special needs school	0.180^*^	−0.156^***^	−0.012
SARS-CoV-2 related variables	SARS-CoV-2 related fears		−0.006^***^	−0.001
	Increased private burdens		−0.090^***^	−0.019^***^
	Increased conflicts household		−0.025^***^	0.011^*^
	Restrictions recreational activities		−0.002	−0.002
	Economic difficulties		−0.090^***^	−0.025^**^
Work-related variables	Work family conflict			−0.154^***^
	Predictability			0.097^***^
	Influence at work			0.118^***^
	Hiding emotions			−0.063^***^
	Emotional demands			−0.053^***^
	Feedback			0.046^***^
	Support colleagues			0.016^*^
	Support supervisors			0.020^**^
	Meaning of work			0.212^***^
	Unfair treatment			−0.048^***^
	Cannot forget work			0.011
	Emotional exhaustion			−0.016^***^
	Adj. *R*^2^	0.022^***^	0.087^***^	0.364^***^
	Degrees of freedom	9	14	26

Unstandardized beta coefficients are reported. ^*^p < 0.05, ^**^p < 0.01, and ^***^p < 0.001. The reference category is in parentheses.

The results of model one revealed a lower job satisfaction in women compared to men (*b* = −0.125). Concerning age, job satisfaction decreases with an increasing average age (*b* = −0.005), the corresponding regression-coefficient was close to zero. In addition, it can be determined that persons who worked in school management showed significantly higher job satisfaction than those who did not (*b* = 0.224). Teachers who worked full-time had a higher job satisfaction than those who worked part-time. Regarding the different types of schools, model one showed that teachers who worked at vocational or special needs schools show higher job satisfaction. In all three models, a large proportion of the sociodemographic variables significantly affected job satisfaction.

In the second regression model, SARS-CoV-2-related variables were added. Theoretically, these variables belong to the burdens domain. The results showed that stronger corona-associated fears (*b* = −0.006), increased private burdens (*b* = −0.090), and economic difficulties (*b* = −0.090) reduced job satisfaction. The restrictions in recreational activities showed no significant regression-coefficient on job satisfaction.

In the third model, variables from the work domain were added. As conflicts between work and private life increased, job satisfaction decreased (*b* = −0.154). Also increased emotional demands (*b* = −0.053), hiding emotions (*b* = −0.063), unfair treatment (*b* = −0.048), and emotional exhaustion (*b* = −0.016) reduced job satisfactions while higher work predictability (*b* = 0.097), influence on the work (*b* = 0.118), feedback (*b* = 0.046), support of colleagues (*b* = 0.016), and supervisors (*b* = 0.020) and a high meaningfulness of work (*b* = 0.212) increase job satisfaction.

The adjusted *R*^2^ was 0.364 in the third regression model (Table 2). Accordingly, the third model explained 36.4% of the variance in job satisfaction. In the first model (adj. *R*^2^ = 0.022) and the second model (adj. *R*^2^ = 0.087), the adjusted *R*^2^ was still relatively small (Schendera, 2014).

## 4. Discussion

The aim of the present study was to assess job satisfaction among teachers in Germany during the SARS-CoV-2 pandemic and to identify relevant predictors of job satisfaction among teachers. Since all variables of the work-related variables (except for emotional exhaustion) were recorded with an equal scale, the following statement can only be formulated for the work-specific variables: a look at all work-related variables showed that work family conflicts, work influence, work meaning, and work predictability, were key predictors of job satisfaction. This can be explained by the fact that the beta coefficients are high, especially for the work-specific variables and connected to this the adjusted *R*^2^ becomes larger due to adding the work-specific variables in model 3. Therefore, it can be noted that especially the work-related variables contributed to the variation in job satisfaction. However, this assumption must be considered with caution, because adjusted *R*^2^ only gives information about the goodness or quality of models with different numbers of predictors and make these comparable. When interpreting, it should be taken into account that unstandardized coefficients were reported, and thus the strongest influence could not be identified across all variables. The increase in influence on the work, feedback, and meaningfulness of work lead to increased job satisfaction.

As shown in model 2, it was again evident that burdens (such as emotional exhaustion or unfair treatment) negatively influenced job satisfaction. The consistently negative coefficients of the SARS-CoV-2-related variables confirmed the associated initial considerations, that burdens, in this case SARS-CoV-2-related problems, worsened job satisfaction. The regression parameters of the different school types decreased with the addition of the other variables. Therefore, it can be stated, that the differences in job satisfaction between the individual types of schools could be a result of the different working conditions especially for vocational and special needs schools. When adding the work-specific variables in model 3, the regression coefficient of gender did not become significant anymore. It can be assumed, that work family conflicts in particular explained most of the variance of a potential gender effect. Descriptive results and other studies confirmed that conflicts between work and private life are more pronounced in women than in men, as they still take on a large part of the care housework despite breaking role models (Doucet, 1995; Craig and Baxter, 2016).

Following on from the present findings on the predictors influencing job satisfaction, there were many other associations and research areas associated with the topic. The complex of topics therefore offers a suitable working point for future studies, which will be further discussed in the conclusion.

## 5. Limitations

During the analysis, a few limitations emerged, which will be discussed below. Due to the very high number of cases in the sample, there were significant regression-coefficients on almost all variables. For this reason, the present results should be viewed with caution regarding their significance. It should also be noted that this is only a cross-sectional study conducted at a specific time during the pandemic. Studies at other times during the pandemic could lead to different results. Another limitation is the dependent variable of job satisfaction. Even though some research literature confirmed that the online questionnaire of job satisfaction with a single item is sufficient (Nagy, 2002; Dolbier et al., 2005), there are also some recommendations to survey job satisfaction using a multi-level scale (Evans, 1997). This could help identify the individual areas of job satisfaction more precisely and analyzed them in more detail. For analyses focusing on the theoretical construct job satisfaction, it makes sense to use the validated item batteries. This would also facilitate the derivation of specific recommendations. It would be easier to identify aspects of a person’s work life with which they are dissatisfied and what possible interventions exist to improve them. Nevertheless, the questionnaire with a single item is also a valid instrument for recording general job satisfaction, especially in the case of many different topics, as in our study. At the time of the online questionnaire, Germany was at the beginning of the third wave. Increasing infection rates and a change to remote teaching could lead to additional uncertainties and a bias in the results. Due to the cross-sectional nature of the study, it is also difficult to draw conclusions about changes compared to before the pandemic.

Moreover, our analysis was purely exploratory, based on basic theoretical frameworks, but does not explain causal effects. This would require a different methodological approach or a precise specification and measurement of relevant predictors. It should also be noted that a large part of the recruitment was done through the Education and Science Union (GEW). Since participation in the GEW is voluntary, this could result in a bias regarding the sample. It should also be noted that we cannot know if mainly motivated and active teachers or those who were not doing well and were highly burdened participated in the online questionnaire.

Although there are some limitations to the study, its strengths should not be overlooked. These include the large number of cases, the fact that the study was conducted nationwide in Germany, and the fact that it covered a wide range of topics. It is therefore a suitable basis for making statements about the situation of teachers throughout Germany during the SARS-CoV-2-pandemic in March 2021 and for illuminating various topics.

## 6. Conclusion and practical recommendations

The present work showed that predictors of job satisfaction could be derived especially from work-related variables. The question posed at the beginning about relevant predictors of job satisfaction during the SARS-CoV-2 pandemic can be answered to the manner that work-related variables, which deal with the structures and organization of daily tasks, were suitable predictors of job satisfaction. In contrast, restrictions or fears related to the pandemic played only a minor role. However, it can be assumed that the stressors caused by the pandemic can also be found in the work characteristics (Petrakova et al., 2021). In particular, the school environment underwent various and often nontransparent changes. A holistic approach should be taken when designing working conditions and establishing firm feedback structures and levels of autonomy. In this context, seemingly fixed structures, such as in the teaching profession, should be reconsidered and adapted to the needs. For example, online training on the use of technology in the classroom, direct feedback opportunities, or times for colleagues to share ideas with one another could help improve their well-being in the school setting. Even if the construct of job satisfaction cannot be used to record psychological burdens, it can be used as an instrument for recording a first impression. Improving job satisfaction can make a significant contribution to promoting teachers’ health (Faragher et al., 2005). Studies showed that the attitudes of school leaders in particular played a decisive role in health promotion programs (Betschart et al., 2022).

Numerous studies showed that job satisfaction also influences the output of work. For example, several studies showed a relationship between job satisfaction and absenteeism (Hoogendoorn et al., 2002; Schaumberg and Flynn, 2017). However, this relationship is controversially discussed in the literature, as the causal direction is partly questionable, and studies found no correlation between job satisfaction and absenteeism (Bridges, 1980). In addition, there was a correlation between job satisfaction and burnout symptoms. In the study by Steinlin et al. (2016), group comparisons showed that people with low job satisfaction had a higher prevalence of burnout symptoms (Steinlin et al., 2016). Based on these results, measuring job satisfaction can help capture the individual health status of respondents without asking personal questions about their mental health.

Further research should focus on capturing the work-specific variables in more detail and look at the respondents’ suitable work environment. In the context of the teacher sample, it would have been interesting to differentiate between lesson preparation, teaching situations, or organizational aspects such as general conferences. In order to continue to make the field of education an attractive professional field in the future, research in the area of schools is particularly recommended. The work family conflict aspect could also be considered further. For example, an attempt could be made to record whether differences in the perception of conflict result from parts of the duration, location, or distribution of working time (Absenger et al., 2014). Specifically, in the case of teachers, it seems interesting to record how much working time is spent preparing and organizing lessons and the daily school routine. From the public health point of view, conditions at the workplace, for example material or technical equipment, as well as preventive interventions to promote health, could also be the subject of further investigation.

Satisfied teachers will also be able to conduct higher-quality lessons and educate children better (Turner and Thielking, 2019). Finally, the topic of job satisfaction offers a wide variety of possible differentiation and is an important construct to capture the well-being at work.

## Data availability statement

The raw data supporting the conclusions of this article will be made available by the authors, without undue reservation.

## Ethics statement

The studies involving human participants were reviewed and approved by Ethical committee of the Medical Association of Rhineland-Palatinate, Germany (application number: 2020-15531). The patients/participants provided their written informed consent to participate in this study.

## Author contributions

CK, VE, PD, TB, and SL: conceptualization. TD, CK, VE, CZ, and PD: data curation. TD, CK, and VE: formal analysis. CK, PD, TB, and SL: funding acquisition and supervision. TD and PD: investigation, validation, visualization, and writing—review and editing, project administration, resources, and writing—original draft. TD, CK, VE, KK, CZ, TB, and PD: methodology. TD, CK, KK, and CZ: software. All authors contributed to the article and approved the submitted version.

## Funding

The project was funded by the Federal Institute for Occupational Safety and Health (BAuA) and with significant support of the Institute of Occupational, Social and Environmental Medicine of the University Medical Center at the University of Mainz. Funding number: F2513.

## Conflict of interest

The authors declare that the research was conducted in the absence of any commercial or financial relationships that could be construed as a potential conflict of interest.

## Publisher’s note

All claims expressed in this article are solely those of the authors and do not necessarily represent those of their affiliated organizations, or those of the publisher, the editors and the reviewers. Any product that may be evaluated in this article, or claim that may be made by its manufacturer, is not guaranteed or endorsed by the publisher.

## Supplementary material

The Supplementary material for this article can be found online at: https://www.frontiersin.org/articles/10.3389/fpsyg.2023.1168647/full#supplementary-material

## Footnotes

^1^Further information about the topics and the establishment procedure of the online questionnaire can be obtained from Koestner et al. (2022).

^2^Through the pretests, 4 previously selected variables were excluded because they did not show significant results.
