# Supplementary material for: Predictors of job satisfaction among teachers in Germany during the SARS-CoV-2 pandemic: cross-sectional results of a nationwide online questionnaire
Source: Front Psychol. 2023 May 26;14:1168647. doi: 10.3389/fpsyg.2023.1168647 (PMC10250674; doi:10.3389/fpsyg.2023.1168647)
Supplement: Supplementary file 1 [file Data_Sheet_1.pdf]

Supplementary Material

Predictors of job satisfaction among teachers in Germany during the SARS-CoV-2 pandemic: cross-sectional results of a nationwide online questionnaire

Theresa Dicks, Viktoria Eggert, Clemens Koestner, Carolina Zähme, Till Beutel, Kristin Kalo, Stephan Letzel, Pavel Dietz*

***** Corresponding Author: pdietz@uni-mainz.de

1 Supplementary Tables

1.1 Table 1: Measurement of work-related variables

**Variable**	**Question**	**Responsescale**
Single items		
Meaning of work	Is your work meaningful?	1 “To a very low degree”, 2 “To a lesser extent”, 3 “To some extent”, 4 “To a great extent”, 5 “To a very high degree”
Social support colleagues	How often do you receive help and support from your colleagues when needed?	1 “Never /almost never”, 2 “Rarely, 3 “Sometimes”, 4 “Often”, 5 “Always”
Social support supervisors	How often do you receive help and support from your immediate supervisor when needed?	1 “Never /almost never”, 2 “Rarely, 3 “Sometimes”, 4 “Often”, 5 “Always”
Cannot forget work	How often can you not forget about work in your free time?	1 “Never /almost never”, 2 “Rarely, 3 “Sometimes”, 4 “Often”, 5 “Always”
Scales		
Work-family conflict	My work takes up so much energy that it has a negative impact on my personal life.	1 “To a very low degree”, 2 “To a lesser extent”, 3 “To some extent”, 4 “To a great extent”, 5 “To a very high degree”
	My work takes up so much time that it has a negative impact on my personal life	1 ”To a very low degree”, 2 “To a lesser extent”, 3 “To some extent”, 4 “To a great extent”, 5 “To a very high degree”
Predictability	Are you informed well in advance about changes in your workplace, e.g. important decisions, changes or plans for the future?	1 ”To a very low degree”, 2 “To a lesser extent”, 3 “To some extent”, 4 “To a great extent”, 5 “To a very high degree”

	Are you getting all the information you need to do your job well?	1 ”To a very low degree”, 2 “To a lesser extent”, 3 “To some extent”, 4 “To a great extent”, 5 “To a very high degree”
Influence at work	Do you have much influence over decisions that affect your work?	1 “Never /almost never”, 2 “Rarely, 3 “Sometimes”, 4 “Often”, 5 “Always”
	Do you have any influence on the amount of work that is assigned to you?	1 “Never /almost never”, 2 “Rarely, 3 “Sometimes”, 4 “Often”, 5 “Always”
	Do you have any influence on what you do at work?	1 “Never /almost never”, 2 “Rarely, 3 “Sometimes”, 4 “Often”, 5 “Always”
Hiding emotions	Does your work require you to hide your feelings?	1 ”To a very low degree”, 2 “To a lesser extent”, 3 “To some extent”, 4 “To a great extent”, 5 “To a very high degree”
	Does your work require you to hide your feelings?	1 ”To a very low degree”, 2 “To a lesser extent”, 3 “To some extent”, 4 “To a great extent”, 5 “To a very high degree”
Emotional demands	Is part of your job to deal with other people's personal problems?	1 ”To a very low degree”, 2 “To a lesser extent”, 3 “To some extent”, 4 “To a great extent”, 5 “To a very high degree”
	Is your work emotionally demanding?	1 ”To a very low degree”, 2 “To a lesser extent”, 3 “To some extent”, 4 “To a great extent”, 5 “To a very high degree”
Feedback	How often does your supervisor talk to you about the quality of your work?	1 “Never /almost never”, 2 “Rarely, 3 “Sometimes”, 4 “Often”, 5 “Always”
	How often do your colleagues talk to you about the quality of your work?	1 “Never /almost never”, 2 “Rarely, 3 “Sometimes”, 4 “Often”, 5 “Always”

1.2 Table 2: KMO & Bartlett’s Test

Kaiser-Meyer-Olkin Measure of Sampling Adequacy.		0.703
Bartlett's Test of Sphericity	Approx. Chi-Square	31003.858
	df	6
	Sig.	.000

1.3 Table 3: Factor loadings of principal component analysis

Component Matrix[a]

	Component
	1
How strong is your fear of being infected with the SARS-CoV-2 virus?	0.848
If you were to contract COVID-19: How likely do you think it is that this disease will take a severe course?	0.649
How strong is your fear of becoming a carrier of the SARS-CoV-2 virus yourself, i.e., infecting others around you with the corona virus?	0.805
How strong is your fear of friends or relatives becoming infected with the SARS-CoV-2 virus?	0.850

Extraction Method: Principal Component Analysis.
a. 1 components extracted.

1.4 Table 4: Spearman correlation of independent variables

	Job satisfaction
Job satisfaction	1
Gender	-.072**
School management	.066**
Working time model	.046**
Primary school	-.052**
Secondary general school	.002
Secondary school	-.037**
Academic secondary school	.027**
Comprehensive school	-.006
Vocational school	.033**
Special needs school	.053**
Other school types	-.003
Age	-.041**
Minor children in household	.009
Work family conflict	-.407**
Predictability	.296**
Influence on the work	.356**
Hiding emotions	-.300**
Emotional demands	-.143**
Feedback	.172**
Support colleagues	.211**
Support supervisors	.261**
Meaning of work	290**
Unfair treatment	-.331**
Cannot forget work	-.155**
Emotional exhaustion	-.422**
SARS-CoV-2 associated fears	-.214**
Increased private burdens	-.187**
Increased conflicts household	-.121**
Restrictions recreational activities	-.043**
Economic difficulties	-.123**

** Correlation is significant at the 0.01 level (2-sided).
